# Construction of antibiotic-induced depression mice model and the function of intestinal microbiota

**DOI:** 10.3389/fmicb.2023.1093486

**Published:** 2023-11-27

**Authors:** Handan Deng, Yongjun Yu, Qi Sha, Weiyi Sun, Lundan Liang, Feier Ren, Hua Ji, Xiangdi Shen, Xingli Fan

**Affiliations:** ^1^School of Basic Medicine and Forensic Medicine, Hangzhou Medical College, Hangzhou, Zhejiang, China; ^2^School of Clinical Medicine, Hangzhou Medical College, Hangzhou, Zhejiang, China

**Keywords:** antibiotic, depression, intestinal microbiota, depressive-like behavior, neurobiological factors, metagenomics, fecal microbiota transplantation

## Abstract

Many research studies focus on intestinal microbiota-related depression induced by the usage of antibiotics, but the use of antibiotics is fairly different. To construct an effective antibiotic-induced depression mice model and explore the effect of intestinal microbiota in antibiotic-induced depression, we used several kinds of antibiotic mixtures to induce mice depression and used depression-related behavioral tests and neurobiological factors to evaluate the construction of the antibiotic-induced depression mice model. SPSS statistical software was used to analyze the above data, and the optimal model was selected according to the stability of the results and the simplicity of the modeling methods. Metagenomic analysis and fecal microbiota transplantation (FMT) of intestinal microbiota from antibiotic-induced depression mice were performed to analyze the effect of intestinal microbiota. The results showed that antibiotic mixture A (1.25 μg/mL natamycin, 5 mg/mL neomycin sulfate, and 5 mg/mL bacitracin), antibiotic mixture B (24 mg/mL bacitracin, 24 mg/mL neomycin sulfate, 9.6 mg/mL ampicillin, 4.8 mg/mL meropenem, and 1.47 mg/mL vancomycin), and antibiotic solution D (only containing 5 mg/mL neomycin sulfate) could induce depression-like behavior in mice. By using these antibiotics, the concentrations of norepinephrine (NE), 5-hydroxytryptamine (5-HT), and brain-derived neurotrophic factor (BDNF) in mice hippocampus and prefrontal cortex tissues were significantly decreased. All the above results were consistent with those of chronic unpredictable mild stress (CUMS) depression mice. The FMT results showed that fecal microbiota from antibiotic-induced depressed mice transplanted into normal mice (8 weeks-old male C57BL/6J SPF mice) also could induce depression-like behavior and cause similar changes in neurobiological factors. Metagenomic analysis showed that the community structure of microbiota in the intestinal tract of antibiotic-induced depression mice was significantly different from that in control mice, the intestinal microbiota species diversity in antibiotic-induced depression mice was lower, the lipoic acid metabolism pathway was significantly activated, and the abundance of functional gene *lipA* was explicitly increased. Quantitative real-time PCR (qPCR) further verified the abundance of enriched bacteria in the intestinal microbiota of antibiotic-induced depression mice. In summary, the specific antibiotic mixtures can induce depression by causing changes in intestinal microbiota in mice. Antibiotic-induced depressed mice show differences in intestinal microbiota abundance, high enrichment of the unique metabolic pathway, and the functional gene.

## 1 Introduction

Depression is a common mental disease that mainly manifests as persistent low mood, lack of interest, anhedonia, and self-destruction tendencies. With the characteristics of low recovery and high recurrence, it seriously affects human health and the quality of life (Malhi and Mann, 2018), and the pathogenesis of depression is still not clear (Fiksdal et al., 2019; Zhang et al., 2019; Xu et al., 2020; Suneson et al., 2021). The clinical studies and animal experiments on depression showed that depression is always accompanied by dysfunction of the neurotransmitter system and hypothalamic-pituitary-adrenal (HPA) axis, such as the decreased concentrations of norepinephrine (NE), 5-hydroxytryptamine (5-HT), and brain-derived neurotrophic factor (BDNF) in the hippocampus and prefrontal cortex (PFC) tissue (Chandley et al., 2013; Kelly et al., 2016; Emon et al., 2020; Song et al., 2020; Bakusic et al., 2021; Deng et al., 2021) and the increased concentration of adrenocorticotropic hormone (ACTH) and cortisol (CORT) in the serum (Barden, 2004; Lamers et al., 2013; Fiksdal et al., 2019).

According to recent research, the intestinal microbiota is related to the occurrence and development of many diseases, especially depression, through the microbiota-gut-brain axis (MGBA) (Kelly et al., 2016; Cryan et al., 2019; Chevalier et al., 2020; Mörkl et al., 2020; Zhuang et al., 2020). Clinical research also found that the constitution of intestinal microbiota in depression patients was quite different from that in healthy people (Jiang et al., 2015; Shen et al., 2021). The intestinal microbiota may play an important role in the occurrence and development of depression.

Antibiotics are commonly used as clinical bactericidal drugs. Antibiotic use can cause short-term or long-term changes in the intestinal microbiota composition, increasing the risk of depression (Bercik et al., 2011; Guida et al., 2018). The above research suggests that antibiotic use can cause depression by inducing changes in intestinal microbiota. Therefore, many animal studies focused on the depression induced by using antibiotics which caused changes in the intestinal microbiota (Fröhlich et al., 2016; Leclercq et al., 2017; Murphy et al., 2018). This suggests that antibiotic use can cause depression by inducing changes in the intestinal microbiota.

However, in previous relevant studies, there were various problems, such as more types of antibiotics used in modeling, longer modeling cycle, and the possibility of stress reaction caused by gavage in mice (Guida et al., 2018; Hao et al., 2021; Wang et al., 2021). At the same time, in our previous study, we also successfully induced mice depression by using antibiotic mixtures (Fan et al., 2022). It was interesting that two same antibiotics appeared in two antibiotic mixtures that could successfully induce mice depression: neomycin sulfate and bacitracin. Therefore, in this study, we added some new antibiotic mixtures to explore the effects of the two antibiotics, in order to find a simple but effective and stable antibiotic-induced mice depression model.

## 2 Materials and methods

### 2.1 Antibiotic mixture preparation

Antibiotic mixtures were prepared based on document literature and our previous research (Fan et al., 2022). Antibiotic mixture A (AMA) contained 1.25 μg/mL natamycin, 5 mg/mL neomycin sulfate, and 5 mg/mL bacitracin. Antibiotic mixture B (AMB) contained 24 mg/mL bacitracin, 24 mg/mL neomycin sulfate, 9.6 mg/mL ampicillin, 4.8 mg/mL meropenem, and 1.47 mg/mL vancomycin. Antibiotic mixture C (AMC) contained 5 mg/mL neomycin sulfate and 5 mg/mL bacitracin. Antibiotic mixture D (AMD) contained only 5 mg/mL neomycin sulfate. Antibiotic mixture E (AME) contained only 5 mg/mL bacitracin. All the antibiotics were bought from Macklin Co., Ltd., Shanghai, China.

### 2.2 Animals and treatment

Eight-weeks-old male C57BL/6J mice were provided by Hangzhou Paisiao Biological Technology Co., Ltd. The mice were randomly divided into 8 groups with 10 mice in each group. Mice of group AMA were given solution AMA instead of pure water for 7 days, and mice of groups AMB and AMC were given gavage solution AMB and AMC (0.2 mL/10 g/day) for 7 days, respectively. Mice of groups AMD and AME were given solution AMD and AME instead of pure water for 14 days. CUMS depression mice (group CUMS) were established by administering the following stress stimuli randomly once a day for 3 weeks as a positive control according to our previous research (Fan et al., 2022): ① overnight lighting; ② light and dark cycle reversed; ③ tilting the cage at 45° for 7 h; ④ fasting for 18 h; ⑤ water deprivation for 18 h; ⑥ wet cage for 7 h; ⑦ binding for 30 min; ⑧ swimming in 4°C ice water for 5 min. The group of mice gavage normal saline (0.2 mL/10 g/day) for 7 days was used as the negative control (group NS). Mice of the control group were allowed *ad libitum* access to food and water.

### 2.3 Behavioral test

Behavioral tests of the open field test (OFT), tail suspension test (TST), forced swimming test (FST), and sucrose preference test (SPT) were used to observe the depressive behavior of mice after the end of antibiotic treatment (Fan et al., 2022). In the OFT, mice were placed in an open field of 50 cm × 50 cm × 40 cm for 5 min, and the time stayed in the central area was recorded. In the TST, mice were suspended approximately 2 cm from the tip of the tail for 6 min, and the immobility time in the last 4 min was recorded. In the FST, mice were placed in a swimming cylinder (21 cm height × 12 cm internal diameter) filled with water (25°C ± 1°C) and forced to swim for 6 min, and the immobility time for the last 4 min was recorded. In the SPT, on the first day, two bottles of 1% sucrose solution were placed into each cage. After 24 h, one bottle with pure water was replaced. After 12 h, the positions of the two bottles were switched. After 24 h, each mouse was separated from the cage and began to abstain from food and water. After 24 h, a bottle of 1% sucrose solution and 1 bottle of pure water were weighed and then placed into each cage. The consumption of sucrose solution and pure water for each mouse was calculated 3 h later. Sucrose preference rate = sucrose solution consumption/total fluid consumption × 100%.

### 2.4 Neurobiological factor detection

The concentrations of BDNF, NE, and 5-HT in the hippocampus and PFC tissue of each group of mice were detected using an ELISA kit (Meimian, Wuhan, China), after the behavior tests. The concentration of ACTH and CORT in the serum of each group of mice was detected using an ELISA kit (eLGbio, Guangzhou, China). Specific practical steps were followed as per the corresponding kit instructions.

### 2.5 Metagenomic analysis

Fresh feces of mice were collected after antibiotic treatment. According to the results of the behavior tests and the neurobiological factor detection, metagenomic analyses were used to identify the intestinal microbiota changes in depressive mice. Bacterial DNA was extracted by using cetyltrimethylammonium bromide. After confirmation of DNA qualities, sequencing libraries were generated using NEBNext@ UltraTM DNA Library Prep Kit for Illumina (NEB, United States) following the manufacturer’s recommendations, and index codes were added to attribute sequences to each sample. The clustering of the index-coded samples was performed on a cBot Cluster Generation System according to the manufacturer’s instructions. After cluster generation, the library preparations were sequenced on an Illumina NovaSeq 6000 platform, and paired-end reads were generated. The raw data of bacteria, fungi, and viruses were obtained by metagenomic sequencing using the Illumina NovaSeq high-throughput sequencing platform. Kraken2 and the self-build microbial database (sequences belonging to bacteria, fungi, archaea, and viruses were screened from the NT nucleic acid database and RefSeq whole-genome database of NCBI) were used to identify the species contained in the samples, and then, Bracken was used to predict the actual relative abundance of species in the samples. Linear discriminant analysis effect size (LEfSe) was performed to find the characteristic microorganisms of each group. The characteristic metabolic pathway and functional gene were analyzed based on the Kyoto Encyclopedia of Genes and Genomes Pathway Analysis (KEGG) and KEGG Orthologous Groups (KO) databases. Meanwhile, the observed species index, Chao1 index, and Shannon index were used to evaluate the α-diversity of intestinal microbiota. At the operational taxonomic unit (OTU) level, β-diversity was estimated by computing the Bray–Curtis distance and visualized using principal coordinate analysis (PCoA).

### 2.6 Quantitative real-time PCR

The whole bacterial DNA was extracted using the Stool DNA Extraction Kit (Omega Bio-Tek, United States). The qPCR was used to detect 16S rRNA gene abundance of certain bacteria enriched in intestinal microbiota antibiotic-induced depression mice to verify the results of the metagenomic analysis. Universal 16S rRNA was to evaluate the total bacteria abundance. All primers are presented in Table 1 (Tong et al., 2011; Chia et al., 2020; Tsai et al., 2021). All experiments were repeated more than three times. The bacterial absolute abundances were analyzed by utilizing the total bacterial abundance (Jian et al., 2020).

**Table 1:** Primers used for real-time PCR analysis of lncRNA levels.

Gene name	Forward primer (5′ to 3′)	Reverse primer (5′ to 3′)
Universal 16S rRNA	TCCTACGGGAGGCAGCAGT	GGACTACCAGGGTATCTAATCCTGTT
16S rRNA gene of *Akkermansia muciniphila*	CAGCACGTGAAGGTGGGGAC	CCTTGCGGTTGGCTTCAGAT
16S rRNA gene of *Bacteroides thetaiotaomicron*	GCAAACTGGAGATGGCGA	AAGGTTTGGTGAGCCGTTA
16S rRNA gene of *Anaerostipes caccae*	GCGTAGGTGGCATGGTAAGT	CTGCACTCCAGCATGACAGT

### 2.7 Fecal microbiota transplantation

To determine whether the intestinal microbiota plays a crucial role in antibiotic-induced mice depression, the fecal microbiota was suspended from the feces of the mice with depressive behavior and depressive-related changes in neurobiological factors. The feces, which were collected after antibiotic treatment and frozen at −80°C, were selected and 200 mg was added to 5 mL of normal saline, shaken and mixed for 3 min, and left standing for 2 min. Then, the upper layer of bacterial solution was taken as suspended microbiota. The suspended microbiota solution was transplanted into normal 8 weeks-old male C57BL/6J SPF mice (randomly divided into 10 mice per group) by intragastric administration (Li et al., 2019; Zhang et al., 2020). FMTA, FMTB and FMTD group mice were gavaged with fecal microbiota suspensions from AMA, AMB and AMD group mice, respectively, for 2 weeks (0.2ml every other day) (Li et al., 2019; Zhang et al., 2020). The mice gavage normal saline for 2 weeks was used as a negative control.

### 2.8 Statistical analysis

All the data were analyzed using SPSS statistical software, version 23.0. Homogeneity tests and analysis of variance (ANOVA) were performed in this study. Dunnett’s multiple comparison test was used after the homogeneity of variance test. The adjusted *p*-value of less than 0.05 was considered statistically significant.

## 3 Results

### 3.1 Behavioral tests after antibiotic treatment

**Figure 1:**
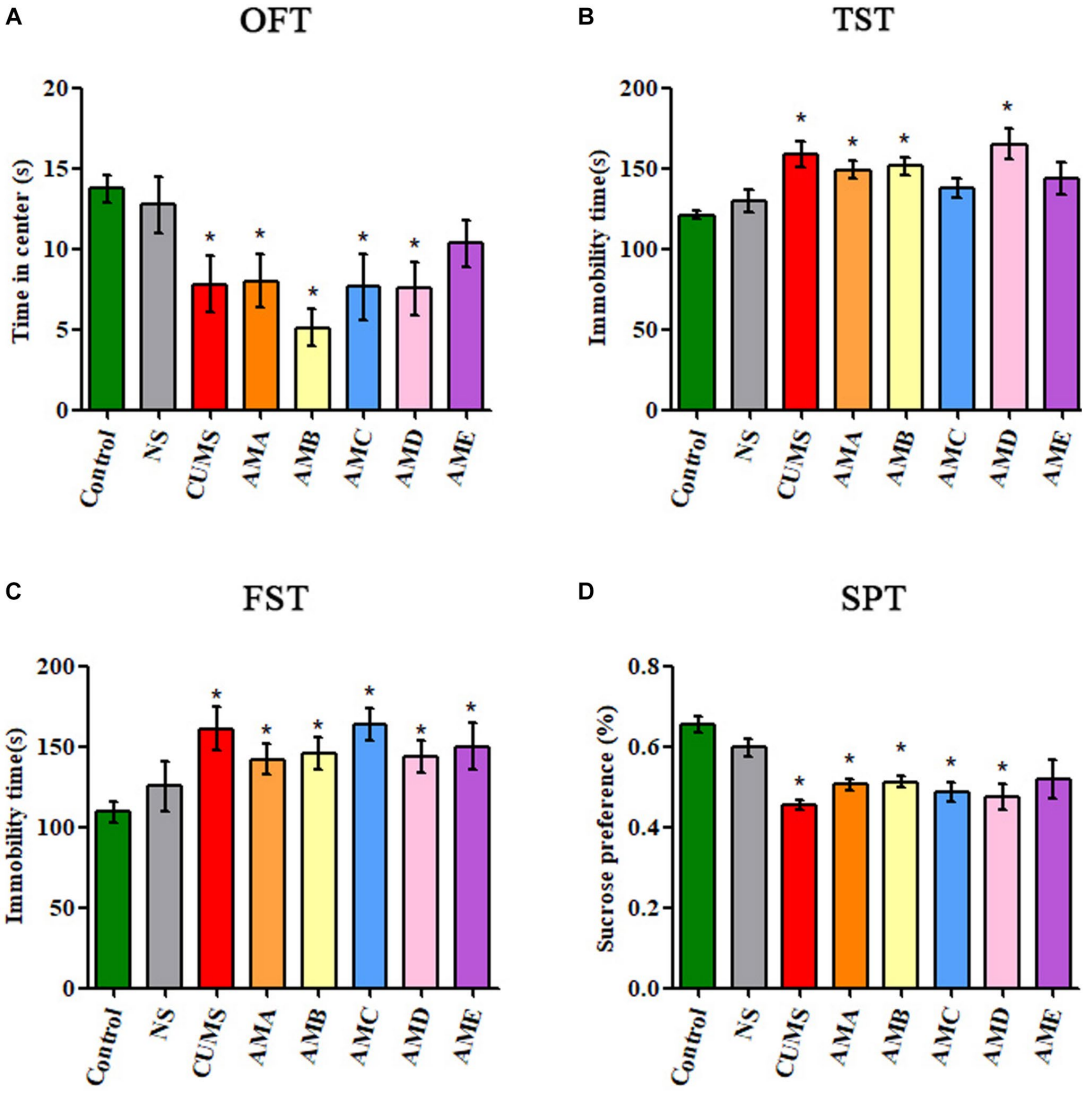
Behavioral test results of antibiotic-treated mice (AMA, AMB, AMC, AMD, and AME), CUMS depression mice (CUMS), control mice, and normal saline gavage mice (NS). **(A)** The residence time of mice in the central area of OFT. **(B)** The immobility time of mice in TST. **(C)** The immobility time of mice in FST. **(D)** The sucrose preference of mice in SPT. Data are presented as mean ± SEM (*n* = 10). ^*^Indicating a statistically significant difference (*p* < 0.05).

Compared with the control group, mice in groups AMA, AMB, and AMD showed depressive behavior: reduced residence time in the open field central area (*p* < 0.05, Figure 1A), increased immobility time in TST and FST (*p* < 0.05, Figures 1B,C), and significantly decreased sucrose preference (*p* < 0.05; Figure 1D), which was the same as the mice with CUMS depression.

### 3.2 The neurobiological factor concentration of mice after antibiotic treatment

**Figure 2:**
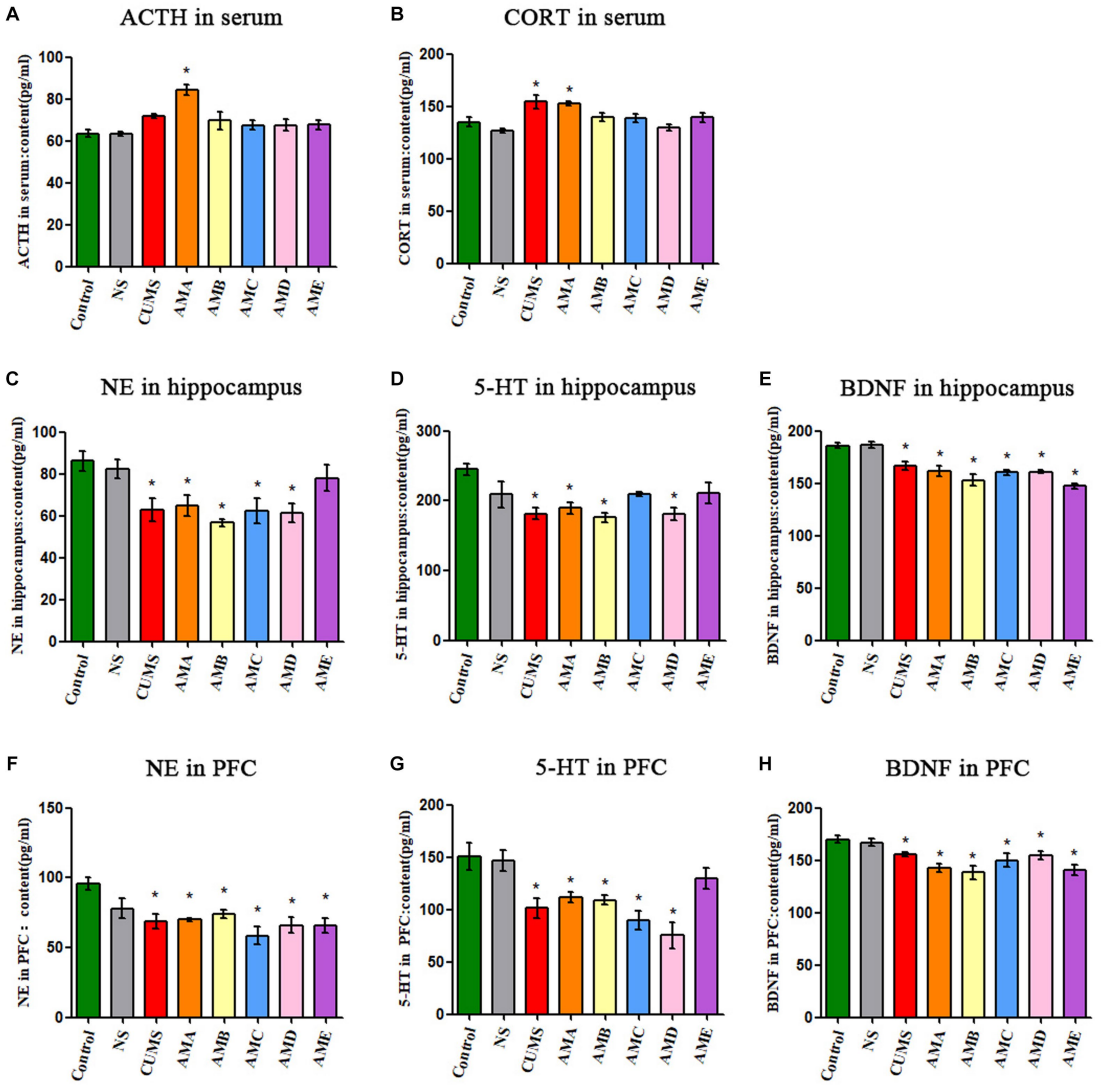
Neurobiological factor detection results of mice. **(A)** The ACTH serum concentration of mice. **(B)** The CORT serum concentration of mice. **(C)** The content of NE in the hippocampus of mice. **(D)** The content of 5-HT in the hippocampus of mice. **(E)** The content of BDNF in the hippocampus of mice. **(F)** The content of NE in the prefrontal cortex of mice. **(G)** The content of 5-HT in the prefrontal cortex of mice. **(H)** The content of BDNF in the prefrontal cortex of mice. Data are expressed as mean ± SEM (*n* = 9). ^*^Indicating a statistically significant difference compared with control mice (*p* < 0.05).

Compared with the control group, the concentrations of ACTH and CORT in serum of group AMA mice were significantly increased (*p* < 0.05, Figures 2A,B). The concentrations of NE, 5-HT, and BDNF in the hippocampus, and PFC tissues of group AMA, ABM, and AMD mice were significantly decreased (*p* < 0.05; Figures 2C–H), and it also showed the same changes in CUMS depression mice.

### 3.3 The metagenomic analysis results of antibiotic-treated mice with depressive behavior

**Figure 3:**
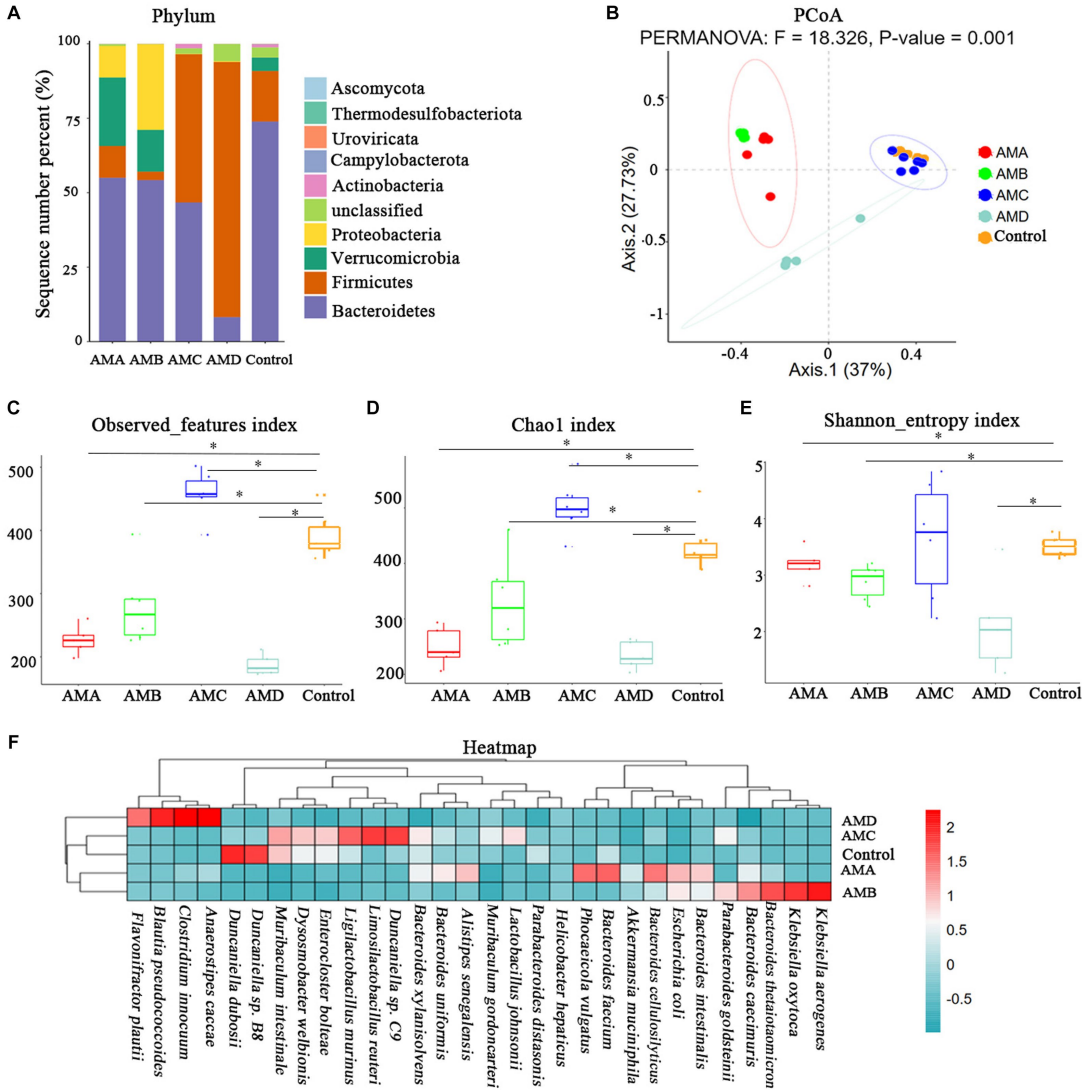
Metagenomic analysis of intestinal microbiota in antibiotic-induced depression mice (AMA, AMB, and AMD) and control mice. **(A)** The microbiota composition of intestinal microbiota at the phylum level of different groups of mice. **(B)** PCoA of intestinal microbiota β diversity among different groups of mice at the OTU level. **(C)** The observed index of microbiota among different groups of mice. **(D)** The Chao1 index of microbiota among different groups of mice. **(E)** The Shannon index of microbiota among different groups of mice. **(F)** Heatmap of bacteria species in the intestinal microbiota of the different groups of mice. All the relative abundances of bacteria were standardized by the *z*-score method. “0” meant the average relative abundance, the values larger than 0 indicated higher abundance, and the values smaller than 0 indicated lower abundance. The color gradient represents the relative abundance of bacteria, ranging from high (2, dark red) to low (−0.5; dark green). Data are expressed as mean ± SEM (*n* = 6). ^*^Indicating a statistically significant difference compared with control mice (*p* < 0.05).

The intestinal microbiota of group AMA, AMB, AMC, and AMD mice and the control group mice were mainly composed of Bacteroidetes, Firmicutes, Verrucomicrobia, Proteobacteria, and Actinobacteria at the phylum level (Figure 3A). PCoA showed that the community structure of the intestinal microbiota in group AMA, AMB, and AMD mice was quite different from that in control mice except group AMC. However, the community structure of intestinal microbiota in group AMA and AMB mice was similar (Figure 3B). The intestinal microbiota species diversity in group AMA, AMB, and AMD mice was lower than in the control group (*p* < 0.05, Figures 3C–E). In addition, relative species abundance was significantly different in antibiotic-induced depression mice from control mice. *Akkermansia muciniphila*, *Bacteroides faecium*, *Phocaeicola vulgatus*, and *Escherichia coli* were the characteristic bacteria species in group AMA; *Bacteroides thetaiotaomicron*, *Klebsiella oxytoca*, *Bacteroides caecimuris*, *Klebsiella aerogenes*, and *Escherichia coli* were the characteristic bacteria species in group AMB; and *Blautia pseudococcoides*, *Clostridium innocuum*, *Anaerostipes caccae*, and *Flavonifractor plautii* were the characteristic bacteria species in group AMD. Compared with the above three groups, the bacteria enriched in group AMC were *Duncaniella* sp. *C9*, *Limosilactobacillus reuteri*, *Enterocloster bolteae*, *Dysosmobacter welbionis*, and *Muribaculum intestinale*, which were more similar to the bacteria enriched in the control group (*p* < 0.05, Figure 3F).

**Figure 4:**
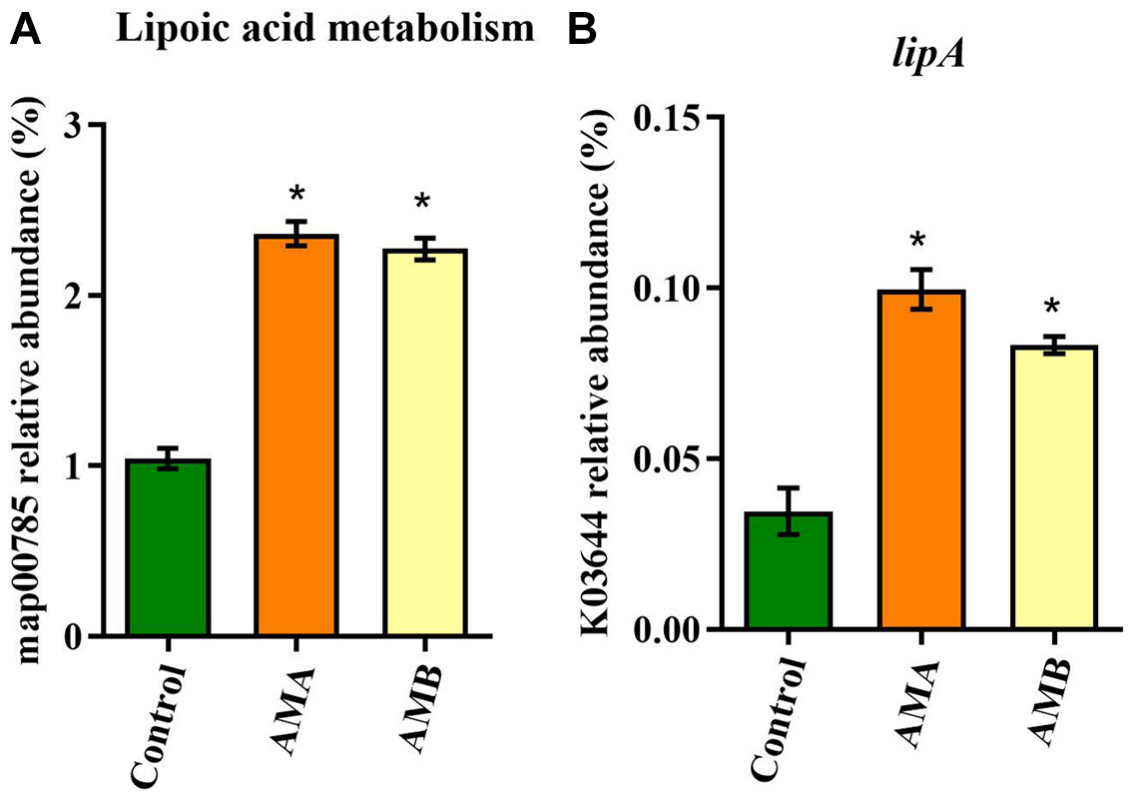
Characteristic metabolic pathway and KO gene of intestinal microbiota from antibiotic-induced mice (AMA and AMB). **(A)** The characteristic metabolic pathway in antibiotic-induced depression mice. **(B)** The typical functional KO gene *lipA* in antibiotic-induced depression mice. Data are expressed as mean ± SEM (*n* = 6). ^*^Indicating a statistically significant difference compared with control mice (*p* < 0.05).

Based on the KEGG and KO databases, compared with control mice, the lipoic acid metabolism pathway (map00785) was highly enriched in group AMA and AMB mice (Figure 4A). The lipoyl synthase gene *lipA* (K03644) in this pathway was the characteristic KO gene in group AMA and AMB mice (Figure 4B).

### 3.4 The results of qPCR

**Figure 5:**
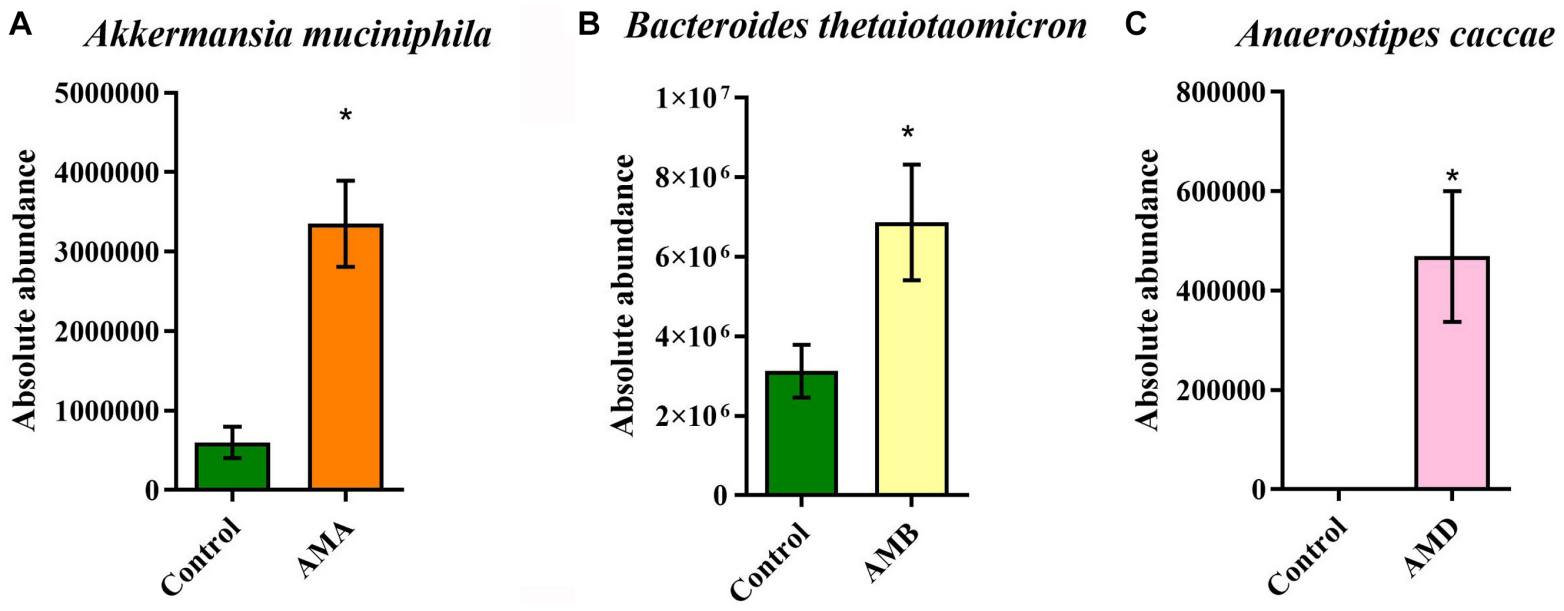
Abundance of enriched bacteria in each group. **(A)** The relative abundance of *Akkermansia muciniphila* in the control group and group AMA. **(B)** The relative abundance of *Bacteroides thetaiotaomicron* in the control group and group AMB. **(C)** The relative abundance of *Anaerostipes caccae* in the control group and group AMC. Data are expressed as mean ± SEM (*n* = 5). ^*^Indicating a statistically significant difference compared with the control group (*p* < 0.05).

The results of qPCR showed that the abundances of *Akkermansia muciniphila* in group AMA mice, *Bacteroides thetaiotaomicron* in group AMB mice, and *Anaerostipes caccae* in group AMD were significantly increased (*p* < 0.05; Figures 5A–C), compared with the control group. The results were consistent with those of metagenomic analysis.

### 3.5 The behavioral test results of mice after FMT

**Figure 6:**
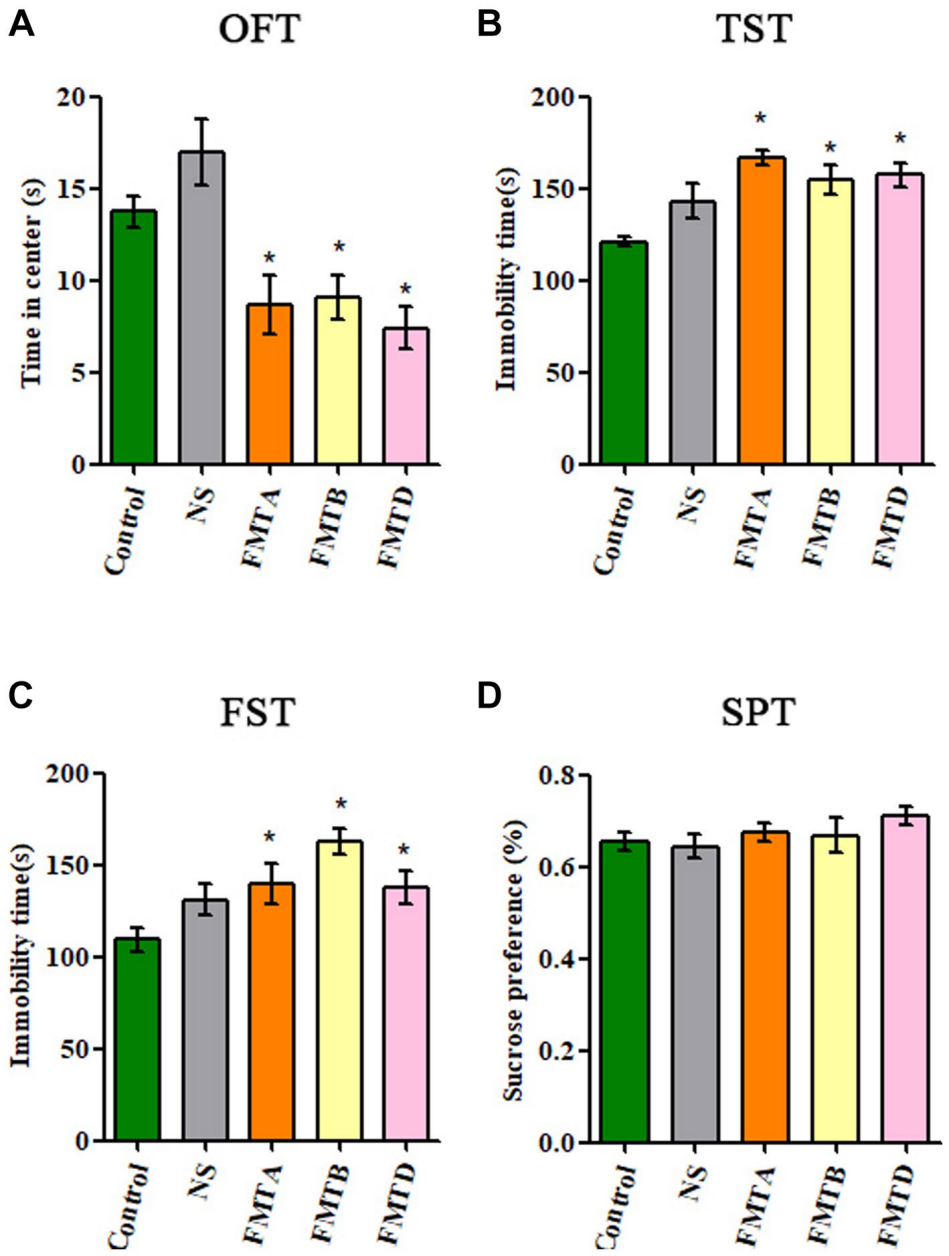
Behavioral test results of mice after FMT. **(A)** The residence time of mice in the central area of OFT. **(B)** The immobility time of mice in TST. **(C)** The immobility time of mice in FST. **(D)** The sucrose preference of mice in SPT. Data are expressed as mean ± SEM (*n* = 10). ^*^Indicating a statistically significant difference compared with the control group (*p* < 0.05).

Compared with control mice, the central area residence time in the OFT of group FMTA, FMTB, and FMTD mice was significantly reduced (*p* < 0.05; Figure 6A), and the immobility time in TST and FST of group FMTA, FMTB, and FMTD mice was increased significantly (*p* < 0.05; Figures 6B,C). However, the preference for sucrose had no significant difference between the FMT mice and control mice (Figure 6D).

### 3.6 The neurobiological factors concentration of mice after FMT

**Figure 7:**
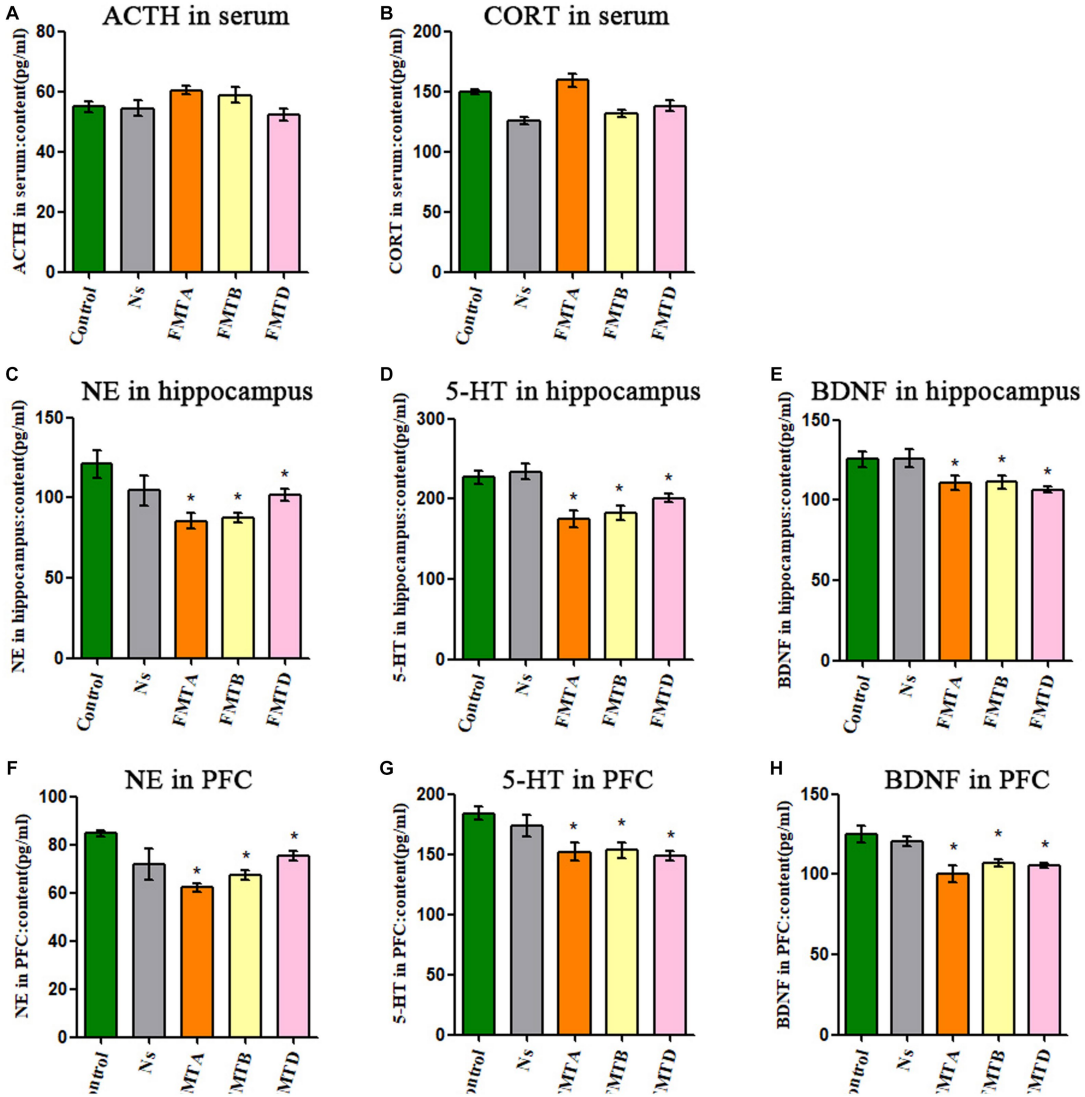
Neurobiological factor detection results of mice after FMT. **(A)** The ACTH serum concentration of mice. **(B)** The CORT serum concentration of mice. **(C)** The content of NE in the hippocampus of mice. **(D)** The content of 5-HT in the hippocampus of mice. **(E)** The content of BDNF in the hippocampus of mice. **(F)** The content of NE in the prefrontal cortex of mice. **(G)** The content of 5-HT in the prefrontal cortex of mice. **(H)** The content of BDNF in the prefrontal cortex of mice. Data are expressed as mean ± SEM (*n* = 9). ^*^Indicating a statistically significant difference compared with control mice (*p* < 0.05).

The concentration of ACTH and CORT in serum had no significant difference between the FMT mice and control mice (Figures 7A,B). Still, the concentration of NE, 5-HT, and BDNF in the hippocampus and PFC in group FMTA, FMTB, and FMTD mice was significantly decreased, compared with the control mice (*p* < 0.05; Figures 7C–H).

## 4 Discussion

The CUMS depression mice model is a recognized and mature animal model widely used in depression-related research (Huang et al., 2019; Qian et al., 2021; Zhang et al., 2021). According to recent research, antibiotic abuse may increase the risk of depression. Dysbacteriosis caused by antibiotic abuse might contribute to the pathogenesis of depression. More and more researchers study the pathogenesis of depression caused by dysbacteriosis based on antibiotic use. However, there is no uniform, stable, and recognized antibiotic-induced depression mice model.

Depression is affected by complex factors, and the mechanism is still unclear. At present, research on pathogenesis mainly focuses on the following aspects, insufficient secretion of NE and 5-HT (Moret and Briley, 2011; Bhatt et al., 2021; Lu et al., 2021), increased secretion of ACTH and CORT caused by abnormal HPA axis (Mayer et al., 2018; Hirtz et al., 2021), and the insufficient secretion of neurotrophic factors (Shimada et al., 2014; Jiang et al., 2018; Guan et al., 2021). Therefore, depressive behavior tests and depression-related factors such as ACTH, CORT, NE, 5-HT, and BDNF were usually used to evaluate the depression mice model.

In our previous study, we successfully induced mice depression by using two kinds of antibiotic mixtures (Fan et al., 2022). It was interesting that neomycin sulfate and bacitracin both appeared in the antibiotic mixtures mentioned above which could successfully induce mice depression. In addition, it has been found that intestinal flora is specifically sensitive to neomycin which can affect the social behavior of mice (Wu et al., 2021), suggesting the importance of neomycin in the study of MGBA. Therefore, in this study, we added some new antibiotic mixtures (neomycin solution, bacitracin solution, and a mixture of these two) to confirm whether the above two antibiotics have an effect on mice depression, in order to find a simple, effective, and stable antibiotic-induced mice depression model.

The mice of the antibiotic-induced groups AMA, AMB, AMC, AMD, and AME showed different degrees of changes in depression-like behavior. However, in view of relevant studies, the contents of BDNF, NE, and 5-HT in the hippocampus and prefrontal cortex of depressed individuals are reduced (Abdel-Bakky et al., 2021; Rana et al., 2021), and the contents of ACTH and CORT in the serum are increased (Mannel et al., 1997; Choi et al., 2018). Therefore, according to the results of neurobiological factor detection and depression-like behavior, the effects of antibiotic-induced depression of antibiotic mixtures C and E were not as efficient as the effects of antibiotic mixtures A, B, and D.

The antibiotic mixtures of A, B, and D could induce mice depressive behavior and depression-related neurobiological factors changes (including the decrease of NE, 5-HT, and BDNF contents in the hippocampus and prefrontal cortex), which is consistent with the changes in CUMS mice and depressed individuals in related studies. Compared with antibiotic mixtures A and B, antibiotic mixture D only contained neomycin sulfate, and the method of administration was free drinking instead of intragastric administration; it was simpler and less interference and was more suitable for establishing an antibiotic-induced mice depression model.

Studies have shown that antibiotic-induced depression may be closely related to intestinal microbiota changes (Carabotti et al., 2015; Yu et al., 2020; Sovijit et al., 2021). To determine the role of intestinal microbiota, metagenomic analysis of intestinal microbiota was used in this study. According to the results of the metagenomic analysis, the intestinal microbiota of antibiotic-induced depression mice was quite different from control mice. The intestinal microbiota α-diversity of antibiotic-induced depression mice was decreased significantly, as reported previously (Humbel et al., 2020; Tian et al., 2022). Current studies have shown that *Erysipelatoclostridium* and *Clostridium innocuum* abundance in the intestines of depressed patients is significantly higher than that of healthy controls (Zorkina et al., 2022). Meanwhile, some studies have shown the role of *Escherichia coli* in inducing depression (Han et al., 2020; Yun et al., 2021). Our study showed that the relative abundance of the intestinal microbiota of depressed mice in each group had significant changes compared with that of the control group, and there were some similarities with the above results, such as the abundance of *Escherichia coli* in the AMA and AMB groups and the abundance of *Clostridium innocuum* in the AMD group were significantly increased. Functional analysis showed that the characteristic bacteria species, metabolic pathways, and KO genes of antibiotic-induced depression mice were quite different from control mice. There were also some similar typical metabolic pathways and KO genes between groups AMA and AMB. Interestingly, mice given the antibiotic mixture AMC (containing neomycin sulfate and bacitracin) and AMD (containing neomycin sulfate) did not show the same performance. The results of metagenomic analysis further verified that the intestinal microbiota composition of AMC mice was significantly different from that of AMD mice. It confirmed what we had supposed—that antibiotic-induced depression in mice is not directly caused by antibiotics but by the changes in intestinal microbiota. In addition, mice of groups AMA, AMB, and AMD, which all had depressive behaviors, also had quite different intestinal microbiota compositions. It suggests that the role of intestinal flora is complex; depression is not caused by the increase or decrease of any certain bacteria but by the overall changes in the structure of the entire bacterial community.

The results of behavioral tests and neurobiological factor detection after FMT showed that the control mice that had accepted FMT had depressive behaviors, and the hippocampus and PFC concentrations of NE, 5-HT, and BDNF had decreased in the antibiotic-induced depression mice. The results of FMT hinted that intestinal microbiota might play a crucial role in antibiotic-induced depression. The FMT mice did not show a decrease in sucrose preference, and the serum concentration of ACTH and CORT of FMT mice also did not increase significantly, which need further research.

## 5 Limitations

Antibiotic depletion of the intestinal microbiota of mice is often performed before the FMT procedure, to optimize colonization of the transplanted microbiota. Given that our results indicated that antibiotic mixtures could induce depression in mice, we did not use antibiotics to deplete the intestinal microbiota in our FMT study, in order to avoid confounding effects from antibiotic exposure. Therefore, FMT was performed on control SPF mice without prior intestinal microbiota depletion. In addition, we did not perform the FMT of the CUMS group, thus the role of the intestinal microbiota in CUMS depression was not clear.

## 6 Conclusion

In summary, antibiotic solution D containing only 5 mg/mL neomycin sulfate, as a simplified method, given to mice instead of pure water for 14 days could induce mice depressive behavior and cause the NE, 5-HT, and BDNF of the hippocampus and PFC depressive-related changes in CUMS depression mice. The intestinal microbiota of antibiotic-induced depression mice differed significantly from control mice, including metabolic pathways and KO genes. The intestinal microbiota played an essential role in antibiotic-induced depression and could induce depression in control mice by FMT, but the mechanism of antibiotic-induced depression is still unclear and needs further research.

## Data availability statement

The datasets presented in this study can be found in online repositories. The names of the repository/repositories and accession number(s) can be found at: NCBI - PRJNA977631.

## Ethics statement

The animal study was approved by Hangzhou Medical College Animal Ethics Committee (Hangzhou, China). The study was conducted in accordance with the local legislation and institutional requirements.

## Author contributions

XF and XS designed and conducted the study. HD, YY, QS, WS, LL, and FR were responsible for the animal experiment. HJ was responsible for the qPCR. HD collected and analyzed the data. HD and XF wrote the manuscript. All authors contributed to the article and approved the submitted version.
